# Mathematical modeling provides kinetic details of the human immune response to vaccination

**DOI:** 10.3389/fcimb.2014.00177

**Published:** 2015-01-09

**Authors:** Dustin Le, Joseph D. Miller, Vitaly V. Ganusov

**Affiliations:** ^1^Department of Microbiology, University of TennesseeKnoxville, TN, USA; ^2^Hope Clinic of the Emory Vaccine Center, Emory University School of MedicineAtlanta, GA, USA; ^3^Department of Mathematics, University of TennesseeKnoxville, TN, USA

**Keywords:** vaccines, T cell responses, mathematical modeling, yellow fever vaccine, smallpox vaccine, antibody-producing cells

## Abstract

With major advances in experimental techniques to track antigen-specific immune responses many basic questions on the kinetics of virus-specific immunity in humans remain unanswered. To gain insights into kinetics of T and B cell responses in human volunteers we combined mathematical models and experimental data from recent studies employing vaccines against yellow fever and smallpox. Yellow fever virus-specific CD8 T cell population expanded slowly with the average doubling time of 2 days peaking 2.5 weeks post immunization. Interestingly, we found that the peak of the yellow fever-specific CD8 T cell response was determined by the rate of T cell proliferation and not by the precursor frequency of antigen-specific cells as has been suggested in several studies in mice. We also found that while the frequency of virus-specific T cells increased slowly, the slow increase could still accurately explain clearance of yellow fever virus in the blood. Our additional mathematical model described well the kinetics of virus-specific antibody-secreting cell and antibody response to vaccinia virus in vaccinated individuals suggesting that most of antibodies in 3 months post immunization were derived from the population of circulating antibody-secreting cells. Taken together, our analysis provided novel insights into mechanisms by which live vaccines induce immunity to viral infections and highlighted challenges of applying methods of mathematical modeling to the current, state-of-the-art yet limited immunological data.

## 1. Introduction

With the invention of novel experimental techniques to track antigen-specific cellular and humoral responses we have gained insights into magnitude and diversity of T cell responses in mice and humans (Altman et al., 1996; Murali-Krishna et al., 1998; Su and Davis, 2013). Imaging and tetramer enrichment techniques have been used to evaluate antigen-specific T cell responses in the whole mouse (Reinhardt et al., 2001; Moon et al., 2007; Obar et al., 2008; Gruta et al., 2010). These and other studies suggest that in major secondary lymphoid organs of mice there is only 10–1500 of naive CD8 or CD4 T cells specific to a given epitope that is translated into precursor frequency of 1–100 per 10^6^ of naive T cells (reviewed in Jenkins and Moon, 2012). A similar precursor frequency of naive T cells was found in humans (Alanio et al., 2010; Jenkins and Moon, 2012).

Studies in mice have found a very consistent picture of the T cell immune response to an antigen (Antia et al., 2005; Obar et al., 2008). Antigen-specific T cells start dividing after 1–2 days following exposure to an antigen, and the number of T cells increases exponentially in the next 4–5 days. After the peak the number of T cells declines exponentially, and within the next 2–3 weeks a relatively constant number of memory T cells is established. Memory CD8 T cells are maintained at a constant level for life of the animal while memory CD4 T cells are slowly lost (Homann et al., 2001).

Because there is a strong correlation between the number of memory T cells generated following an infection and effector T cells present at the peak of the immune response, factors contributing to the magnitude of the effector T cell response have been a subject of debate. Earlier studies proposed that in mice the magnitude of the T cell response is mainly determined by the number of naive epitope-specific T cells or the precursor frequency (Moon et al., 2007; Kotturi et al., 2008; Obar et al., 2008). Another study, however, by tracking T cell response to multiple epitopes of the same virus (influenza) found that precursor frequency is a poor predictor of the peak immune response (La Gruta et al., 2010). It remains unclear which factors determine magnitude of the T cell response to infection or vaccination in humans (Chen et al., 2000). In contrast with studies in mice (De Boer et al., 2001, 2003; Althaus et al., 2007) the temporal kinetics of the T cell responses to viruses in humans have not been analyzed using mathematical models.

Although antibody responses to haptens have been used to form the basis of our understanding of humoral immunity, many quantitative details of B cell responses still remain incompletely understood. Generation of virus-specific antibodies, plasma cells, and memory B cells in spleen and bone marrow of mice has been nicely tracked in a now classical study (Slifka et al., 1998). More recent work also identified rates of B cell division and death during germinal center reaction (Anderson et al., 2009). Yet, quantitative properties of humoral responses to pathogens in humans such as the rate of B cell proliferation, the rate of antibody production and its change over the course of infection remain largely unknown.

In humans, following an immune response to a pathogen causing an acute infection represents a challenge because it is often hard to know when a person was infected and many acute viral infections tend to be of a relatively short duration (Carrat et al., 2008). In contrast, there is a better understanding of the kinetics of cellular and antibody responses to several chronic viruses of humans such as HIV and HCV (McMichael et al., 2010; Perreau et al., 2013; Park and Rehermann, 2014). Vaccination with live viruses represents a convenient way to study human immune responses. In our analysis we focus on the data coming from two recent trials with two highly efficacious vaccines: yellow fever virus (YFV) vaccine 17D (YF-17D) and vaccinia virus (VV) vaccine (Miller et al., 2008; Ahmed and Akondy, 2011). To date, over 540 million doses of YF-17D have been administered, and this vaccine remains one of the safest and effective vaccines in humans (Cottin et al., 2013; Gotuzzo et al., 2013). Vaccinia virus has been used as a vaccine against smallpox. The vaccine is highly efficient and induces life-long humoral immunity and long-term cellular immunity (Hammarlund et al., 2003; Amanna et al., 2007, 2008). We use experimental data on the kinetics of virus-specific CD8 T cell, antibody-secreting cell, and antibody responses to these viruses to obtain insights into mechanisms leading to the generation of efficient immune memory (Miller et al., 2008; Akondy et al., 2009).

## 2. Materials and methods

### 2.1. Experimental data

The data were generated following vaccination of healthy volunteers of age 21-32 with the YF-17D or smallpox vaccines and were described in detail in previous publications (Miller et al., 2008; Akondy et al., 2009). In short, volunteers were vaccinated with 0.5 ml of the YF-17D vaccine. Virus-specific CD8 T cells were identified using tetramer staining (HLA-A2 restricted T cells recognizing NS4B epitope of YFV (Akondy et al., 2009, see **Figure 3B**) or VV^*CLT*^ of VV (Miller et al., 2008, see **Figure 5C**) on days 3, 11, 14, 30, and 90. YFV virus titers were determined as described previously (Akondy et al., 2009) and here the average among all patients was used (Akondy et al., 2009, see **Figure 3B**). VV-specific antibody titers and frequency of antibody-secreting cells were measured on days 0, 7, 14, 21, 28, and 84 after Dryvax immunization. VV-virus specific antibodies were determined as previously described (Newman et al., 2003). Antibody-secreting cells were identified by flow cytometry as CD27^hi^ CD38^hi^ CD3^−^ CD20^lo/−^ PBMCs as described previously (Wrammert et al., 2008).

### 2.2. Mathematical model for CD8+ T cell kinetics

**Figure 1:**
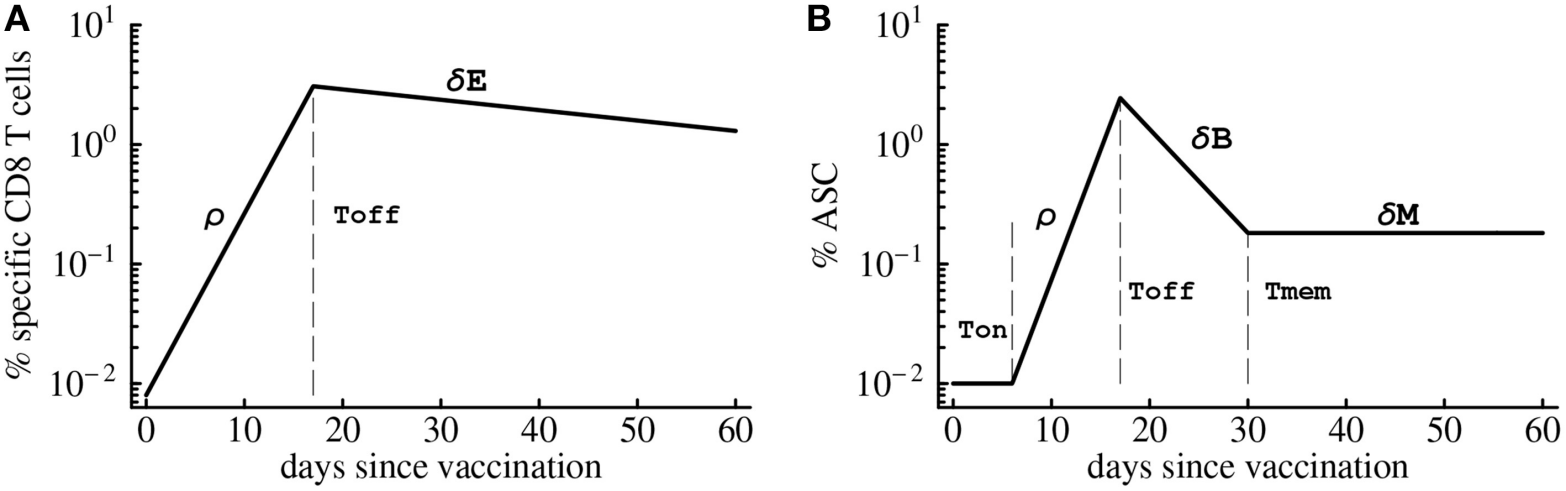
**Schematic cartoon of the kinetics of virus-specific CD8 T cell (A) and antibody-secreting cell (B) responses**. CD8 T cell response is broken down into the expansion phase, peak, and contraction phase. During the expansion phase, CD8 T cells experience massive growth and reach a peak value at *t* = *T*_off_. Once this point is reached, CD8 T cell numbers begin to decline at a rate δ_*E*_. Antibody-secreting cells (ASCs) population starts expanding after the initial delay given by *T*_on_. Similarly as T cells, ASC population grows exponentially at a rate ρ until the peak, declines exponentially after the peak at a rate δ_*B*_ until reaching memory phase at time *T*_mem_. Long-term loss of virus-specific ASCs is given by δ_*M*_ which was set to zero in model fits due to short duration of the experiments.

A simple mathematical model has been previously used to describe kinetics of virus-specific CD8 T cell response in acute and chronic LCMV infection (De Boer et al., 2001, 2003; Althaus et al., 2007). We adopted this model to quantify T cell response in humans (Riou et al., 2012, see Figure 1A). In the model, virus-specific immune response expands exponentially from *E*_0_ precursors at a rate ρ and reaches the peak value *E*_max_ at time *T*_off_. Effector T cells die a constant per capita rate δ_*E*_ (Figure 1A). With these assumptions, the dynamics of the virus-specific CD8 T cell response are given by the following equations:

(1) $$E(t)=\left\{ \begin{array}{l} E_{0}{\text{e}}^{\rho t},\;t<T_{\text{off}},\\ E_{0}{\text{e}}^{\rho T_{\text{off}}\;-\;\delta_{E}(t\;-\;T_{\text{off}})},\;t\geq T_{\text{off}}. \end{array}\right.$$

The model for the dynamics of YFV-specific CD8 T cell response describes accumulation and loss of T cells in circulation. Activation of antigen-specific T cells occurs in secondary lymphoid organs such as lymph nodes or the spleen. Therefore, to determine the impact of the cell dynamics in SLOs on accumulation and loss of activated T cells in circulation we use the following model:

(2) $$\frac{\text{d}E_{L}(t)}{\text{d}t}=\left\{ \begin{array}{l} (\rho_{L}-m_{LB})E_{L}(t),\text{ if }t<T_{\text{off}},\\ -\delta_{E}E_{L}(t),\text{ otherwise}, \end{array}\right.$$

(3) $$\frac{\text{d}E(t)}{\text{d}t}=\left\{ \begin{array}{l} m_{LB}E_{L}(t)-m_{BT}E(t),\text{ if }t<T_{\text{off}},\\ -\delta_{E}E(t),\text{ otherwise}, \end{array}\right.$$

where *E_L_*(*t*) and *E*(*t*) are the density of YFV-specific CD8 T cells in the SLOs and circulation at time *t* since infection, respectively, ρ_*L*_ is the rate of expansion of YFV-specific CD8 T cell population in the SLOs, *m_LB_* is the rate of T cell migration from SLOs into circulation, *m_BT_* is the rate of activated T cell migration from the circulation to tissues during the expansion phase, and δ_*E*_ is the rate of apoptosis of activated YFV-specific CD8 T cells after the peak of the immune response. In the model we assume that cells in circulation do not divide during the expansion phase because we expect that T cells spend only a limited time in circulation (Ganusov and Auerbach, 2014). Including expansion of YFV-specific CD8 T cell response in the blood did not affect the conclusions from the model. During the contraction phase we let cells to die both in SLOs and in circulation, and as the infection is cleared we expect little migration of activated T cells to peripheral tissues.

It should be noted that in this version of the model we assume that activated T cells in circulation do not re-enter SLOs. If the immune response occurs in lymph nodes, the likelihood of lymphocyte re-entry into the same lymph node is low because there are hundreds of LNs in humans (Trepel, 1974). However, if immune response is generated in the spleen, activated T cells in circulation may be able to re-enter this organ. The model that includes generation of the immune response in the spleen and re-entry of activated T cells into the spleen from circulation will be presented elsewhere.

To predict kinetics of yellow fever virus (YFV) growth and clearance we assume that the virus population is growing exponentially and is controlled by the CD8 T cell response which kills virus-infected cells. While we do not know the life-span of free YFV particles, for several viruses such as HIV and HCV, free viral particles are removed very rapidly from circulation (Ramratnam et al., 1999; Guedj et al., 2013), and thus the density of the free viral particles should be proportional to the density of infected cells (Perelson, 2002). Therefore, under the assumption of a rapidly cleared free virus, the dynamics of YFV can be described by the following simple mathematical model:

(4) $$\frac{\text{d}V(t)}{\text{d}t}=V(t)\left(r-\frac{{\left[kE(t)\right]}^{n}}{1+{\left[hE(t)\right]}^{n}}\right),$$

where *V*(*t*) is the density of the virus in the blood (in PFU/ml) in vaccinated individuals at time *t* after infection, *r* is the rate of virus replication, *k* is the efficacy at which YFV-specific CD8 T cells kill YFV-infected cells, 1/h is the percent of the YFV-specific CD8 T cells at which killing of infected cells is half maximal, *n* is the Hill coefficient, and the dynamics of the T cell response is given by Equation (1).

### 2.3. Mathematical model for humoral immune response kinetics

To describe the dynamics of antibody-secreting cells (ASCs) following vaccinia virus (VV) vaccination of human volunteers we extend the model for the CD8 T cell response (see Equation 1) by including an initial delay in the expansion kinetics (*T*_on_) and the memory phase following contraction of the immune response after the peak (Figure 1B). It is well known that antibody-secreting cells produce large amounts of antibodies but it has not been determined whether ASCs in the circulation are responsible for the majority of virus-specific antibodies detected in plasma. To investigate this issue we consider three complementary mathematical models. In the first model, ASCs in the blood produce antibodies at a constant rate and are responsible for the observed levels of virus-specific antibodies in circulation. The dynamics of ASCs (*B*) and antibodies (*A*) are given by the following equations (see also Figure 1B):

(5) $$\frac{\text{d}B(t)}{\text{d}t}=\left\{ \begin{array}{l} 0,\text{ if }t<T_{\text{on}},\\ \rho B(t),\text{ if }T_{\text{on}}\leq t<T_{\text{off}},\\ -\delta_{B}B(t),\text{ if }T_{\text{off}}\leq t<T_{\text{mem}},\\ -\delta_{M}B(t),\text{ otherwise, } \end{array}\right.\;$$

(6) $$\frac{\text{d}A(t)}{\text{d}t}\;=\theta B(t)-\delta_{a}A(t),$$

where θ is the per capita rate of production of antibodies by virus-specific ASCs, and δ_*a*_ is the natural decay rate of antibodies assumed to be δ_*a*_ = 0.0495 day^−1^ corresponding to a half-life of 2 weeks (Slifka et al., 1998; Kindt et al., 2007). The impact of the antibody decay rate δ_*a*_ on our results is outlined in the Discussion Section.

In the second model we assume that ASC and antibody dynamics follow the same model (Equation 5-6) with the rate of antibody production increasing with time since immunization at a rate α,

(7) $$\theta \left(t\right)=\left\{ \begin{array}{l} \theta_{0},\text{ if }t\;<T_{\text{on}},\\ \theta_{0}+\alpha (t-T_{\text{on}}),\text{ otherwise}. \end{array}\right.$$

The dynamics of antibodies in circulation is then given by the equation

(8) $$\frac{\text{d}A(t)}{\text{d}t}=\theta (t)B(t)-\delta_{a}A(t),$$

where the dynamics of ASCs is given by Equation (5). Increase in antibody production with time may occur because of transition of antibody-producing cells from plasmablasts to long-lived plasma cells. Although it was suggested by several experts in the field, we were unable to find direct experimental evidence for the increase in the antibody production rate by ASCs over the course of infection.

Finally, in the third model we allow ASCs in the circulation to differentiate into long-lived plasma cells which then migrate to peripheral tissues such as bone marrow. Such production of long-lived plasma cells in the bone marrow has been well documented following LCMV infection of mice (Slifka et al., 1998). In this model, naive B cells differentiate into antibody-secreting cells (*B*) and the number of ASCs increases exponentially. Following the peak of the response, ASCs die at a rate δ_*B*_ or migrate at a rate *m* to the bone marrow where they rapidly differentiate into long-lived plasma cells (*M*). In the model, virus-specific antibodies in circulation (*A*) accumulate due to production by ASCs in the circulation and by plasma cells in the bone marrow; the rate of antibody production by these cells is θ. The dynamics of circulatory ASCs (*B*), long-lived plasma cells in the bone marrow (*M*), and antibodies in the blood (*A*) are then given by equations:

(9) $$\frac{\text{d}B(t)\;}{\text{d}t}=\{\begin{array}{ll} 0, & \text{if }t<T_{\text{on}},\\ \rho B(t) & \text{if }T_{\text{on}}\leq t<T_{\text{off}},\\ -(\delta_{B}+m)B(t), & \text{if }T_{\text{off}}\leq t<T_{\text{mem}},\\ -\delta_{M}B(t), & \text{otherwise}, \end{array}$$

(10) $$\frac{\text{d}M(t)}{\text{d}t}\;=\{\begin{array}{ll} 0, & \text{if }t<T_{\text{off}},\\ mB(t), & \text{if }T_{\text{off}}\leq t<T_{\text{mem}}, \end{array}$$

(11) $$\frac{\text{d}A(t)}{\text{d}t}\;=\theta (B(t)+M(t))-\delta_{a}A(t).$$

### 2.4. Statistics

The data and model predictions were log-transformed to allow for normally distributed residuals. Data on the YFV-specific CD8 T cell response were fit using the NLS routine in statistical package R. We also used non-linear mixed effects (NLME) routine from R to investigate the importance of variability in model parameters to reproduce immune response kinetics. For the NLME routine, the data from all subjects were taken into account. Non-linear least squares (NLS) routine was used to fit mathematical models to data from subjects that possessed measurements for at least five time points. The system of differential equations were solved using deSolve routine in R. The quality of the model fits to data was compared using either *F*-test for nested models or Akaike Information Criterion, AIC (Bates and Watts, 1988; Burnham and Anderson, 2002). Confidence intervals for the parameters were estimated using standard intervals from nls/nlme routines in R. Fits of the models to the kinetics of antibodies and ASCs following VV vaccination was done in Mathematica 5.2.

## 3. Results

### 3.1. Expansion rate of CD8 T cell responses determines peak magnitude

**Figure 2:**
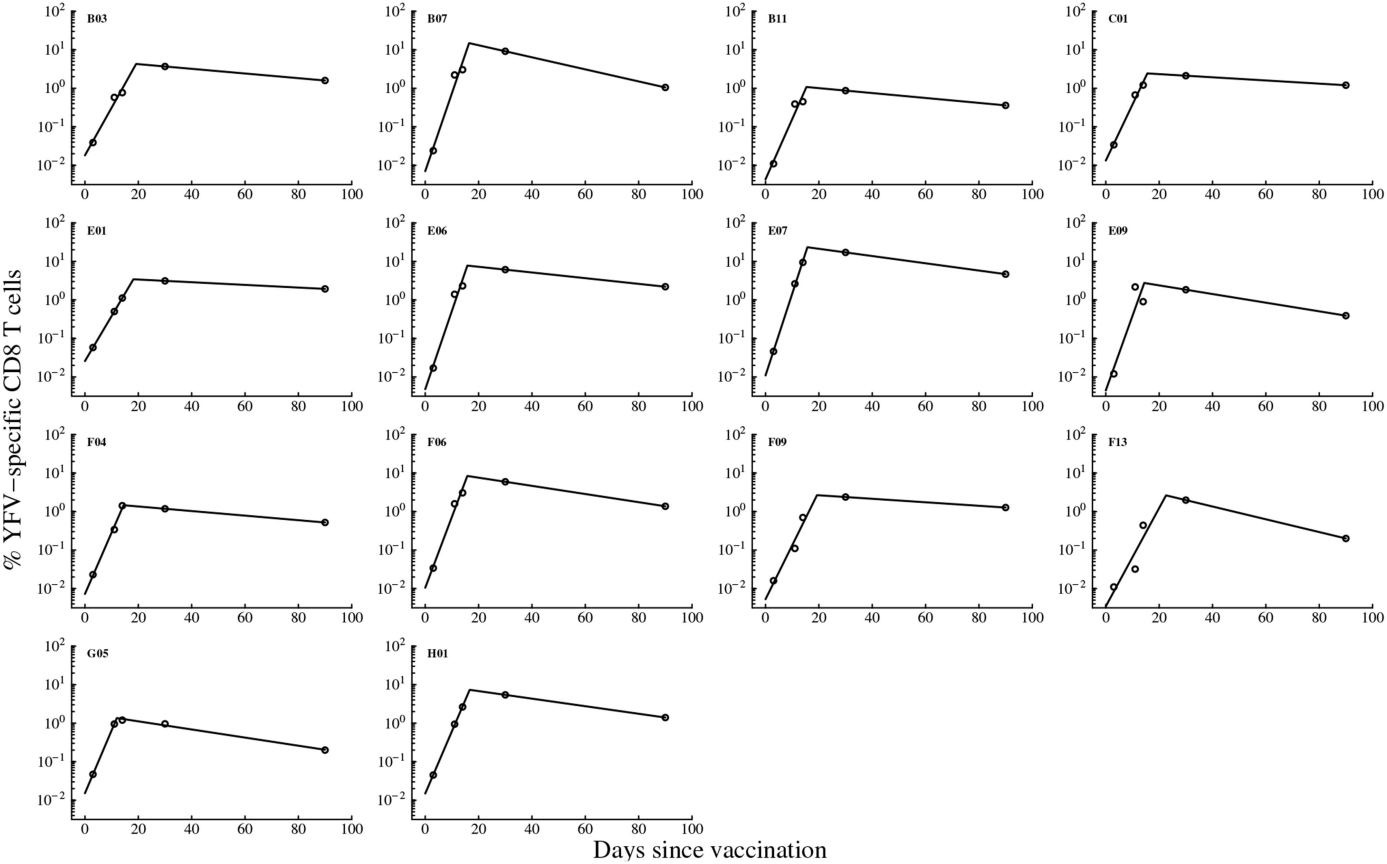
**Kinetics of the YF-17D-specific CD8 T cell response in vaccinated volunteers and predictions of the mathematical model**. We fit the mathematical model given in Equation (1) (see Materials and Methods) to experimental data on the percent of virus-specific CD8 T cells in blood of volunteers and estimate model parameters. Only subjects with at least 5 measurements were included in the analysis because smaller number of data points did not allow estimation of model parameters and associated errors using non-linear least squares. Data are shown by circles and model predictions by lines. Individual panels are data from different subjects with patient labels shown at the top left corner. Estimated parameters of the model are shown in Table 1.

To investigate which parameters determine the magnitude of the antigen-specific CD8 T cell responses in humans, we analyzed kinetic data from a recent trial of Yellow Fever vaccine (YF-17D) in human volunteers (Miller et al., 2008; Akondy et al., 2009). We fitted a mathematical model (see Equation 1 and Figure 1A) to the data on kinetics of HLA-A2-restricted CD8 T cells recognizing NS4B epitope of YF-17D (Akondy et al., 2009) and estimated parameters of the model (Figure 2). Despite being extremely simple, the mathematical model describes experimental data well (Figure 2). Following immunization, YF-17D-specific CD8+ T cells proliferated rapidly, and the model predicts the rate of exponential increase in the total size of the virus-specific CD8 T cell response of ρ = 0.37/day (or doubling time of 1.8 days, Table 1).

**Table 1:** **Model parameter estimates**.

**Patient**	***E*_0_, %**	**ρ, day^−1^**	***T*_off_, day**	**δ_E_, day^−1^**	***E*_max_, %**
B03	0.0182 [0.00004–0.069]	0.28 [0.13–0.50]	19.2 [10.3–28.1]	0.014 [0.001–0.07]	4.3
B07	0.0070 [0.00001–0.027]	0.47 [0.30–0.67]	16.4 [11.6–21.2]	0.036 [0.010–0.08]	14.9
B11	0.0043 [0.00001–0.017]	0.36 [0.20–0.57]	15.3 [09.1–21.6]	0.015 [0.001–0.07]	1.1
C01	0.0133 [0.00003–0.051]	0.33 [0.18–0.54]	15.6 [08.7–22.4]	0.010 [0.007–0.08]	2.4
E01	0.0259 [0.00005–0.099]	0.27 [0.12–0.48]	18.2 [08.9–27.4]	0.008 [0.011–0.08]	3.4
E06	0.0048 [0.00001–0.018]	0.47 [0.30–0.67]	15.8 [10.9–20.6]	0.017 [10^−6^–0.07]	7.8
E07	0.0109 [0.00002–0.042]	0.49 [0.32–0.69]	15.7 [11.1–20.3]	0.022 [0.001–0.07]	23.3
E09	0.0045 [0.00047–0.013]	0.45 [0.33–0.58]	14.4 [11.0–17.8]	0.026 [0.007–0.06]	2.8
F04	0.0073 [0.00001–0.028]	0.37 [0.21–0.57]	14.5 [8.50–20.4]	0.014 [0.001–0.07]	1.5
F06	0.0105 [0.00002–0.040]	0.42 [0.26–0.63]	15.8 [10.5–21.0]	0.024 [0.002–0.07]	8.4
F09	0.0053 [0.00001–0.020]	0.32 [0.17–0.53]	19.3 [11.3–27.3]	0.011 [0.005–0.07]	2.7
F13	0.0035 [0.00001–0.013]	0.29 [0.14–0.51]	22.6 [13.3–31.8]	0.038 [0.011–0.08]	2.6
G05	0.0152 [0.00004–0.064]	0.38 [0.16–0.68]	11.9 [06.8–17.1]	0.024 [0.005–0.06]	1.4
H01	0.0149 [0.00003–0.057]	0.37 [0.21–0.58]	16.7 [10.5–22.8]	0.023 [0.001–0.07]	7.3
Mean	0.0104	0.37	16.5	0.02	5.99
Stdev	0.0065	0.07	4.7	0.01	6.26

*We list estimates of parameters of the model given in Equation (1) fitted to the experimental data on the kinetics of YF-17D-specific CD8 T cells in vaccinated volunteers. Listed parameters are *E*_0_ is the predicted initial percent of virus-specific CD8 T cells (calculated as the percent of YFV-specific CD8 T cells out of total CD8 T cell population), ρ is the rate of expansion of virus-specific CD8 T cell response, *T*_off_ is the predicted time of the immune response peak, δ_*E*_ is the rate of decline of the immune response after the peak, and *E*_max_ is the predicted peak percent of the YFV-specific CD8 T cells. Note that *E*_max_ is not directly estimated from the data but it is defined as E_max_ = E_0_e^ρT^^off^ (see Equation 1). We also list 95% confidence intervals (in brackets) as provided by the nls routine in R*.

By assuming that CD8 T cell response is activated at the time of vaccination, we predict that on average, 1 in 10^4^ of naive CD8 T cells in circulation are specific for the NS4B epitope of YFV (Table 1). This is on a higher end of directly estimated precursor frequencies of antigen-specific CD8 T cells in humans (Alanio et al., 2010; Jenkins and Moon, 2012). If T cells are activated later time points after vaccination, the model would predict even higher precursor frequency (results not shown). Importantly, we found that parameters characterizing initial expansion kinetics of the YFV-specific CD8 T cell response, ρ and *E*_0_, were not significantly correlated (*p* = 0.12), suggesting that both parameters independently contribute to the magnitude of the immune response.

Our model allows for a more precise estimate of the timing (*T*_off_) and the value (*E*_max_) of the peak of the YFV-specific CD8 T cell response (Table 1). The model predicts that on average, the immune response peaks at 16.5 days post vaccination and reaches the maximum of 6% of the total CD8 T cells in circulation. Both values are different from parameters determined for murine CD8 T cell responses specific to another acute viral infection, LCMV, where immune responses peak 7–8 days post infection and T cells specific to a single viral epitope can reach 30–50% of the total CD8 T cell pool (De Boer et al., 2001, 2003). Finally, the model predicts the loss of activated YFV-specific CD8 T cells at an average rate δ_*E*_ = 0.02 per day which is equivalent to half-life of 35 days. This is also a much slower decay than that observed for murine CD8 T cells (De Boer et al., 2003). Finally, by knowing the initial and final frequency of YFV-specific CD8 T cells, we estimate that the observed expansion of the CD8 T cell response was achieved in less than 9 divisions. This is surprisingly low since murine CD8 T cells divide > 10 − 12 times (and possibly more than 15 times) during acute viral or bacterial infections (Blattman et al., 2002; Stemberger et al., 2007; Obar et al., 2008; Miller et al., 2008).

**Figure 3:**
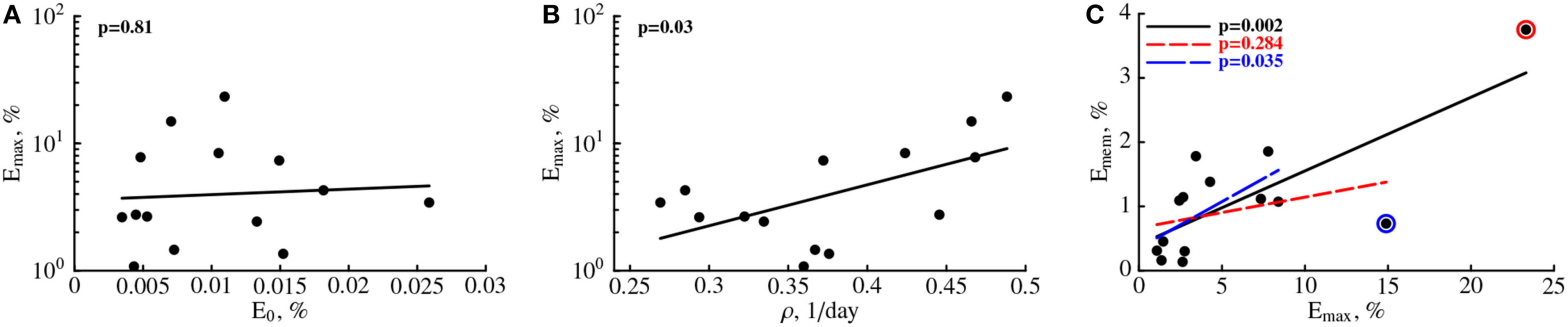
**The rate of expansion of the YFV-specific CD8 T cell response and not precursor frequency predicts peak immune response**. For every patient we plot the predicted peak of the immune response (*E*_max_) vs. estimated precursor frequency *E*_0_ **(A)** or the expansion rate ρ (**B**, see Table 1). Also, we plot a correlation between the frequency of memory CD8 T cells specific to YFV found 100 days post immunization, *E*_mem_ = *E*(100) (see Equation 1), as the function of the predicted peak of the immune response *E*_max_ **(C)**. *P*-values for linear regressions are shown on individual panels. In **(C)** we show regression for the full data (solid line) and the regression with one (small dashing line and red circle) or two (large dashing line and blue circle) outliers removed.

Our analysis allows to investigate which parameters determine the peak of the immune response and the duration of elevated numbers of YFV-specific CD8 T cells. Interestingly, we found that not the initial precursor frequency but the rate of expansion of CD8 T cell response is determining the peak magnitude of the immune response (Figures 3A,B). This is in contrast to several earlier findings in mice where the peak of T cell responses was strongly correlated with the precursor frequency of naive T cells (Moon et al., 2007; Obar et al., 2008; Jenkins and Moon, 2012); our results are more consistent with another study suggesting importance of T cell expansion kinetics in determining immunodominance (La Gruta et al., 2010). To investigate this issue further, we re-fitted our mathematical model to the immune response data using non-linear mixed effects models by allowing only some parameters to vary between individuals. The best fit of the data was obtained with only the expansion rate ρ being varied between subjects (Figure S1 and Table S1 in Supplemental Information). Allowing for variability in precursor frequency between subjects did not improve the quality of the model fit to data further suggesting that precursor frequency did not play the major role in the determining immune response dynamics (*p* = 0.6, likelihood ratio test). Interestingly, the NLME-based fits of the model to data with only expansion rate varied between subjects were significantly better than the fits in which we estimated individual parameters for every immunized volunteer (AIC = 87.1 vs. 109.0 for NLME vs. NLS fits, respectively).

We investigated whether any of the model parameters determine the magnitude of the frequency of YFV-specific memory T cells. Since our measurements did not establish the time when memory T cells are formed, we let the number of activated CD8 T cells found in circulation 100 days post immunization to represent memory T cells. Previous work established that vaccination with vaccinia virus leads to formation of a stable population of memory CD8 T cells by this time (Miller et al., 2008). Interestingly, no individual parameter for the immune response, including the rate of the immune response expansion ρ was predictive of the percent of memory T cells found 100 days post vaccination (*p* > 0.05, results not shown). Interestingly, the estimated peak of the immune response *E*_max_ was not strongly correlated with the memory T cell levels (Figure 3C) since the significance of the correlation was dependent on selection of all or only subset of individuals in the analysis (Figure 3C). The results were similar if we used non-parametric tests for the analysis (results not shown). Furthermore, by random resampling our estimates with replacement we found that in nearly 30% of cases, the correlation between *E*_max_ and *E*_mem_ are not statistically significant at *p* = 0.05 level. The absence of strong correlation between peak immune response and predicted levels of memory T cells are in sharp contrast with studies in mice where such correlation was established (Murali-Krishna et al., 1998).

Finally, we asked which parameters determine the time when the frequency of YFV-specific CD8 T cells declines to a level of naive T cells if the rate of decline of virus-specific CD8 T cells after the peak does not change over time. On average, this time was 378 days. Interestingly, neither the rate of expansion of CD8 T cell response nor the peak immune response was predictive of the time of the immune response loss (results not shown). Rather, the rate of loss of activated CD8 T cells, δ_*E*_, was strongly correlated with the time of the loss of virus-specific T cells (*p* < 0.001). Taken together, our analysis provides novel basic information on the kinetics of CD8 T cell responses to a viral infection in humans and highlight the importance of the parameters determining the rate of immune response expansion and contraction for predicting peak effector response and duration of immune memory.

### 3.2. Viral clearance is correlated with expansion of YFV-specific CD8 T cell response

**Figure 4:**
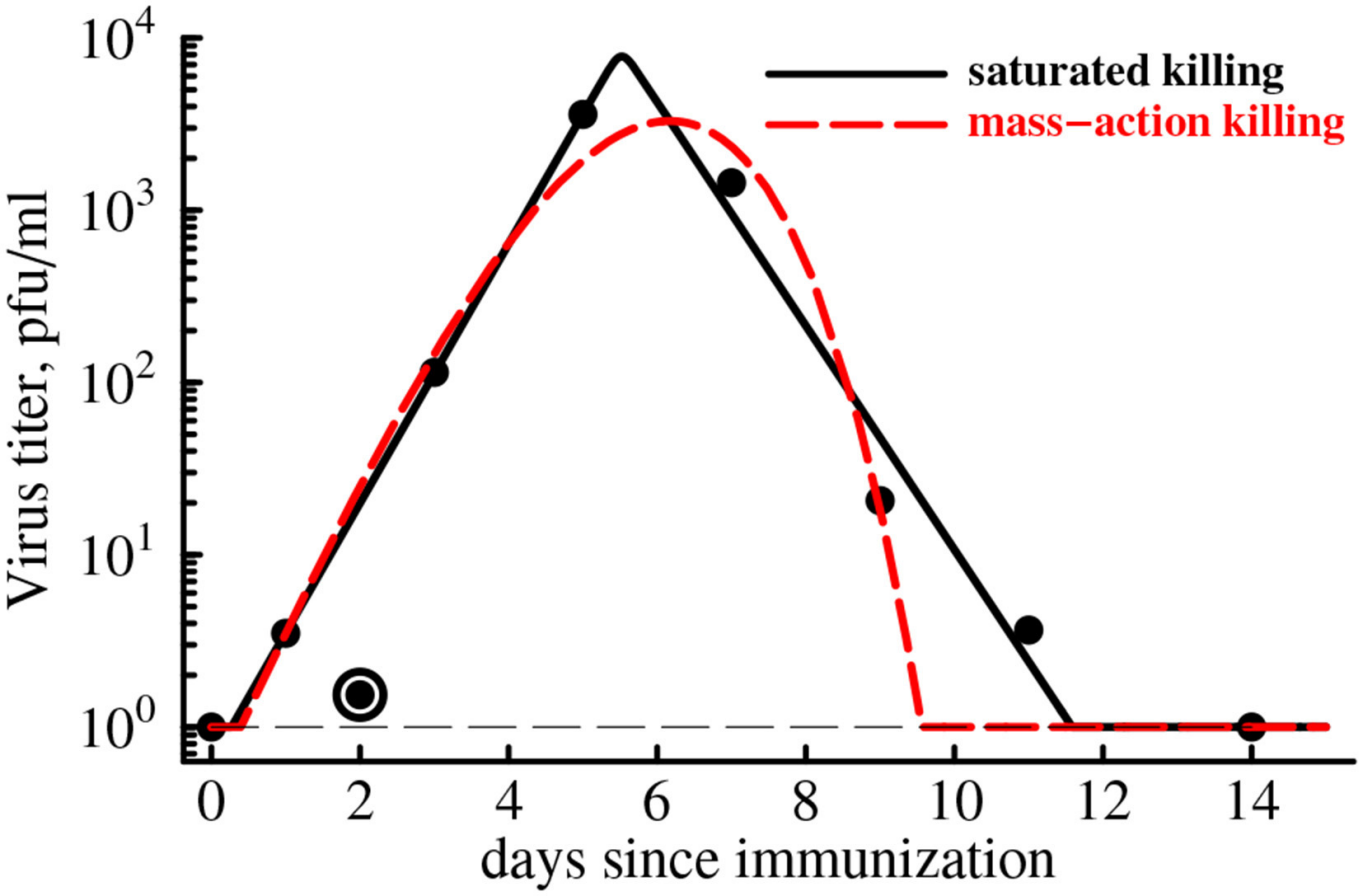
**CD8 T cell expansion kinetics explain clearance of yellow fever virus**. We use parameter estimates for an “average” individual (last row in Table 1) to predict the kinetics of clearance of YFV in the blood with a simple mathematical model (Equation 4). Data are shown as points. Two types of model fits to data are shown: (1) model in which killing of the virus population follows the law of mass-action (*h* = 0, *n* = 1, dashed line), and (2) model in which killing of the virus population is saturated at high CD8 T cell densities (*h* > 0, *n* = 50). We find that the best fit is provided by the model in which killing of virus-infected cells saturates at high density of YFV-specific CD8 T cells even though this improvement in the fit is not statistically significant due to a low number of data points (*F*_1,4_ = 4.94, *p* = 0.09). Parameter providing the best fit are *V*_0_ = 0.43, *r* = 2.39 day^−1^, *k* = 24.4 day^−1^, *h* = 0, *n* = 1 (for the mass-action killing model) and *V*_0_ = 0.63, *r* = 1.73 day^−1^, *k* = 13.3 day^−1^, *h* = 13.0, *n* = 50 (for the saturated killing model). Data point at day 2 was excluded in model fits to the data as an outlier. Hill coefficient was fixed in model fits of the data. The virus detection limit in these experiments was 1 PFU/ml and is shown by a thin dashed line.

YF-17D is a live viral vaccine, and the virus can be easily detected in the blood of vaccinated individuals (Akondy et al., 2009). In the vaccinees, the peak viral load in the blood is reached on average 5 days post immunization, and the virus was cleared within 2 weeks post immunization (Figure 4). Although both cellular and humoral immunity may contribute to viral control in immunized individuals, we set to investigate whether expansion kinetics of the YFV-specific CD8 T cell response is consistent with the clearance of the YFV in circulation. Clearance of several viruses, such as influenza and lymphocytic choriomeningitis virus (LCMV) is correlated with the rise in virus-specific CD8 T cells (Lau et al., 1994; Doherty and Christensen, 2000; De Boer et al., 2001). However, virus-specific CD8 T cell response in mice expand significantly faster than T cell population specific to YFV (De Boer and Perelson, 1998; De Boer et al., 2001; Althaus et al., 2007, Table 1) and therefore we may expect that slowly expanding human YFV-specific T cell responses may not be able to adequately describe viral clearance.

Therefore, we fitted a simple mathematical model for the dynamics of virus to the experimentally measured YFV titers in immunized volunteers (see Equation 4 and Figure 4). In our basic model we assumed that expanding CD8 T cell response clears the virus-infected cells at the rate that is proportional to the density of YFV-infected cells and YFV-specific T cells (so-called mass-action, *n* = 1 and *h* = 0 in Equation 4). Our recent work has shown that killing of peptide-pulsed targets by cytotoxic T lymphocytes in the spleen follows the law of mass action (Ganusov et al., 2011). Interestingly, slowly expanding YFV-specific CD8 T cell response was able to predict rapid viral growth (doubling every 7 h) and viral clearance in less than 2 weeks post infection (Figure 4, dashed line). However, the description of the viral load data was not adequate and viral clearance was predicted to occur 2 days earlier than was actually observed. In the model this occurred because exponentially growing CD8 T cell population was expanding as the virus was being cleared (see Figure 2) resulting in accelerating virus decay after the peak. Furthermore, the model also predicted that YFV-specific CD8 T cells started killing virus infected cells within several days post vaccination because in this model the estimated replication rate of the virus (*r* = 2.4/day) is higher than the actual increase rate of viral titers during this time period (*r*_0_ = 1.7/day was estimated using linear regression of log-transformed viral titers before the peak viremia).

It is possible, however, that killing *in vivo* does not follow the mass-action law, for example, due to competition between killer T cells for the access to infected targets if the number of effectors greatly exceeds the number of virus-infected cells (Ganusov and De Boer, 2008; Graw and Regoes, 2009; Gadhamsetty et al., 2014). The model in which killing of virus-infected cells by the T cell response saturates at high density of effectors more accurately explains the rise and clearance of YFV in vaccinated individuals (Figure 4, solid line). Interestingly, our analysis suggests that for the best description of the data, immune response has “all-or-nothing” effect found at *n* ≫ 1: at low T cell densities, T cells contribute very little to viral control while at high densities, T cell killing reaches the maximum. The exact value of the saturation constant did not play a major role as long as *n* > 10 since models with different values of *n* (*n* ≥ 10) led to fits of a similar quality (results not shown).

Importantly, although the fit of the model with saturated killing leads to a dramatic reduction in the residual sum of squares (from 0.45 to 0.20), the improvement in the fit quality was not statistically significant (*F*_1,4_ = 4.94, *p* = 0.09) most likely due to a limited number of data points. The results were similar if we extend our analysis by using weighted least squares with measured error in viral titers for different time points (results not shown). Extending this analysis to virus and YFV-specific CD8 T cell response kinetics in individual subjects will likely provide additional insights into whether CD8 T cells are responsible for viral clearance in YFV-infected individuals or some other effector mechanisms, e.g., antibody response, must be also involved to explain the data.

In our analysis we assumed that the rate of expansion of the YFV-specific CD8 T cell response is given by the average estimate ρ = 0.37 day^−1^ (Table 1). However, our estimates suggest that the expansion rate could vary substantially between different individuals from 0.12 to 0.69 day^−1^ (including confidence intervals in Table 1). Interestingly, reducing the rate of expansion ρ from 0.37 day^−1^ to lower values did not significantly impact the the quality of the fit of the model with mass-action killing of the data for most values of ρ (results not shown). However, at very low expansion rates, e.g., when ρ = 0.1 day^−1^, the model with mass-action killing was unable to accurately describe the data as judged by significantly increased AIC score (AIC increased by 8.4). Furthermore, increasing the rate of expansion to higher that 0.37 day^−1^ values also revealed poor description of the data by the model with mass-action killing (e.g., at ρ = 0.5 day^−1^ AIC was increased by 12.4). Interestingly, the model with saturated killing (*n* ≫ 1) could still explain the viral clearance data with excellent quality even at lower or higher than 0.37 day^−1^ expansion rates, so that the difference in the fits of the models with mass-action killing vs. saturated killing became statistically significant at ρ = 0.5 day^−1^ (*F*_1,4_ = 22.4, *p* = 0.009). This result suggests that in our models the kinetics of expansion of YFV-specific CD8 T cell response has a strong impact on determining viral clearance kinetics, and that future studies should attempt to determine mechanisms leading to accumulation and loss of antigens-specific CD8 T cells in circulation and possible in tissues of humans.

### 3.3. Kinetics of VV-specific antibody-secreting cells and antibodies

**Figure 5:**
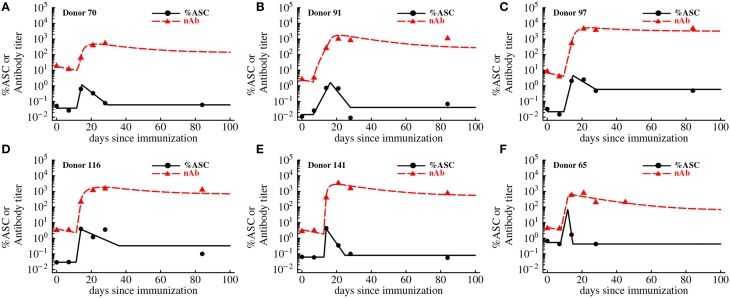
**Kinetics of VV-specific antibodies are predicted well by the accumulation and loss of antibody-secreting cells in the blood**. We fit the basic mathematical model (Equations 5-6, see Materials and Methods for more detail) to the experimental data using non-linear least squares. Data are shown as markers (points are for antibody-secreting cells and triangles are for antibodies), and lines are the predictions of the mathematical model. The data and the fits are shown for individual donors in panels **A–F**, and donor IDs are indicated on individual panels. Best fit parameters are shown in Table 2.

While humoral immune responses to haptens and viruses have been studied for decades, as far as we know there has been no studies that directly linked dynamics of antigen-specific antibody-secreting cells (ASCs) and antigen-specific antibodies in humans in one quantitative framework. Quantification of the antibody production by individual ASCs has been undertaken previously using a technique of isolating individual plasma cells (Jerne and Nordin, 1963; Jerne et al., 1974); yet, the rates of division of ASCs and the rate of antibody production by ASCs *in vivo* in humans remain to be determined. To provide initial answers to these questions we analyzed experimental data on the dynamics of ASCs and antibodies specific to vaccinia virus (VV) in VV-infected individuals (Miller et al., 2008, Figure 5). A low level of virus-binding antibodies was detected in these individuals, and VV-specific antibody titer increased 2-3 orders of magnitude within a week after 1 week delay following vaccination. The frequency of ASCs in circulation also increased following vaccination (Figure 5). To investigate how ASC and antibody dynamics are linked we developed a mathematical model (see Equations 5-6 in Materials and Methods). The model was fitted to experimental data and the best fits are shown in Figure 5 as lines.

In the basic model we assume that after an initial delay, ASCs start proliferating and produce antibodies at a constant rate. Surprisingly, this simple model was able to accurately capture the dynamics of both antibody-producing cells and VV-specific antibodies over 3 months after vaccination (Figure 5). The model predicted that on average immune response starts expanding 10 days post immunization at the average rate of 1.2 per day (doubling time of 14 h) which appears to be a substantially faster expansion than that of YFV-specific CD8 T cells (Table 1) or VV-specific CD8 T cells (see Discussion). The peak of the immune response occurs at 14 days post immunization which is slightly earlier than that of YFV-specific CD8 T cells. We found that the dynamics of VV-specific antibodies can be explained well if ASCs in circulation produce antibodies at a rate *p* = 185 relative units (RU) per 1% of ASCs in circulation per day. In other words, 185 RU of VV-specific antibodies are produced by 1% of ASCs in circulation per day. To convert these estimates to the rate of antibody production by individual cells we will need to know the total number of antibody-producing cells and total amount of antibodies in circulation; these values were not available from the original publications (Miller et al., 2008; Akondy et al., 2009).

Although the mathematical model describes the data reasonably well, some fits are not completely satisfactory; for example in Figures 5B–D, the model underestimates the antibody titers 84 days post immunization. Furthermore, some estimated parameters appear somewhat unrealistic, e.g., rapid expansion rate ρ in the donor 141 (Table 2). Therefore, we tested two additional mathematical models that relax some assumptions regarding ASC dynamics (see Materials and Methods).

**Table 2:** **Parameter estimates determining the kinetics of VV-specific antibody-secreting cells and antibodies**.

**Donor**	***B*_0_, %**	***T*_on_, day**	**ρ, day^−1^**	***T*_off_, day**	**δ_*B*_, day^−1^**	***T*_mem_, day**	***A*_0_, RU**	**θ, day^−1^**
70	0.037	11.6	1.17	14.53	0.2	29.5	17.5	108.6
91	0.015	6.6	0.48	16.39	0.32	28.0	2.5	288.2
97	0.022	9.5	1.02	14.79	0.15	28.0	6.7	261.3
116	0.029	11.3	1.77	14.0	0.11	36.0	4.0	98.8
141	0.062	12.7	3.13	14.0	0.36	24.9	3.5	311.0
65	0.537	7.7	1.20	11.84	1.73	14.8	5.0	7.0
Median	0.033	10.4	1.19	14.3	0.26	28.0	4.5	185.0

*The best fits of the model to experimental data are shown in Figure 5. Here *B*_0_ is the pre-immunization level of ASCs, *T*_on_ is the time of the start of proliferation of VV-specific ASCs, ρ is the rate of expansion of the VV-specific ASC population, *T*_off_ is the time of the peak of ASC response, δ_P_ is the rate of loss of ASCs from circulation, *T*_mem_ is the time when ASC density stops declining, *A*_0_ is the antibody titer prior to vaccination, and θ is the rate of antibody production by VV-specific ASCs. We assume that VV-specific IgG antibodies have a natural half-life time of 2 weeks which translates into antibody removal rate of δ_a_ = 0.0495 day^−1^ (Slifka et al., 1998). This value was fixed in fitting of the model to experimental data*.

In the first alternative model (Equation 5, 8), we allow ASCs to increase the rate of antibody production during an immune response. It is possible that late ASCs make more antibodies on a per cell basis than early ASCs, although we did not find a direct experimental evidence for this hypothesis. Allowing for a linear increase in antibody production rate by ASCs over time allowed for more reasonable description of accumulation and loss of VV-specific antibodies and for smaller estimates of the expansion rate of the ASC population (results not shown). However, because of the limited number of data points and increased number of model parameters, this improvement in the quality of the model fit to data was not statistically significant (*p* > 0.2 for all donors, *F*-test for nested models). In the second alternative model (Equations 9-11) we allowed ASCs in the circulation to migrate to tissues (e.g., bone marrow) and produce antibodies from that distant site. It is well known that following an immune response the majority of virus-specific ASCs in mice are found in the bone marrow (Slifka et al., 1998), and in humans the bone marrow harbors a large population of plasma cells (Turesson, 1976). This extended model also allowed for a better description of the dynamics of antibody titers and predicted that about 10–20% of circulating ASCs migrate to the tissues during the contraction phase of the immune response (results not shown). Yet, the model fits of the data were not statistically improved (*p* > 0.15, *F*-test for nested models). Future studies by providing more frequent measurements of the ASCs and antibodies are likely to improve our quantitative understanding of humoral immune responses to vaccination and may allow to reject the simple model (that assumes that most of antibodies in circulation at 90 days post immunization come from ASCs in circulation).

## 4. Discussion

Basic features of the immune responses to viruses, causing acute infections, appear to be similar in mice and humans. Antigen-specific lymphocytes are activated shortly after the infection; activated lymphocytes proliferate for 7–20 days. After reaching the peak of the immune response many activated cells die and a smaller population of resting lymphocytes survive. For CD8 T cell responses, approximately 5–10% of effector T cells present at the peak of the immune response survive into memory (Kaech and Cui, 2012); memory T cells provide protection following re-exposure to the same infection (Mbow et al., 1994; Co et al., 2002; Miller et al., 2008; Santos et al., 2008). In contrast, during humoral immune response effector lymphocytes (ASCs) survive into the memory and become long-lived plasma cells (Slifka et al., 1998).

While basic features of the virus-specific immune responses are well understood, parameters determining the kinetics of immune responses in humans are not known. In particular, only recently the frequency of human naive T cells specific to several different antigens has been identified (Jenkins and Moon, 2012). Our analysis extends previous work by providing quantitative estimates of the parameters defining kinetics of CD8 T cells, ASCs, and antibodies specific to live viral infections of humans.

We found that the population of CD8 T cells specific to NS4B epitope of YFV expands relatively slowly with the average doubling time of 2 days reaching the peak of the immune response 2.5 weeks post immunization (Figure 2). Analysis of the additional data on the kinetics of VV-specific CD8 T cell response in one patient revealed a similar expansion kinetics at a rate ρ = 0.44 day^−1^ (doubling time of 1.6 days) with the observed peak 2 weeks post infection (results not shown and Miller et al., 2008). This is in sharp contrast with the kinetics of the virus-specific CD8 T cell responses in mice where T cell response expands at a much faster rate (ρ = 2−3 day^−1^, doubling time every 6–8 h) and peaking 7–8 days post infection (De Boer and Perelson, 1998; De Boer et al., 2001). The rate of loss of activated T cells after the peak is also slower in humans than in mice (0.02–0.04 per day vs. 0.20 per day in humans and mice, respectively). Interestingly, the estimated rates for human volunteers are relatively close to the rates of expansion and contraction of the CD8 T cell response against simian immunodeficiency virus in macaques (Davenport et al., 2004). In accord with some but not all studies we found that the peak frequency of the YFV-specific CD8 T cells is mainly determined by the rate of expansion of T cell response (La Gruta et al., 2010). Finally, we found that the predicted peak of the immune response in humans did not correlate significantly with the frequency of memory T cell formed which is also in contrast with observations in mice (Doherty and Christensen, 2000).

It should be emphasized, however, that kinetic parameters for the T cell response in mice have been obtaining using data from secondary lymphoid organs such as spleen, while our analysis is for the T cells found in circulation. We have developed a simple model in which proliferation of antigen-specific CD8 T cells occurs in secondary lymphoid organs (SLOs) at a rate ρ_*L*_; activated T cells leave SLOs into the circulation at a rate *m_LB_*, and cells in the blood migrate to peripheral tissues at a rate *m_BT_* (see Equations 2-3 in Materials and Methods). Direct solution of this mathematical model suggests that after an initial delay the density of virus-specific CD8 T cells in the blood increases exponentially at a rate ρ = ρ_*L*_ − *m_LB_* (result not shown). Therefore, the rate of increase in the density of YFV-specific T cells in the circulation is proportional to that of T cells in SLOs; however, the small value for the expansion rate ρ for the YFV- and VV-specific CD8 T cells may not represent slow division of these cells but rather a balance between cell division in SLOs and the cell exit into circulation. The kinetics of lymphocyte exit from SLOs during an immune response in humans are not known, and in fact, as far as we know, the rates of exit of activated T cells from lymph nodes or the spleen have not been quantified even in mice. We have recently estimated that thoracic duct lymphocytes, which consist mostly of resting T cells, spend on average 2.5 h in the spleen and 10 h in the lymph nodes or Peyer's patches (Ganusov and Auerbach, 2014). Inflammation did not impact the residence time of these cells in lymph nodes. Our unpublished work also suggests that blood-derived lymphocytes spend 20–30 h in ovine lymph nodes in the absence of inflammation (McDaniel and Ganusov, ms. in preparation). If activated T cells spend about 1 day in human lymph nodes (*m*_LB_ ≈ 1 day^−1^), then our simple mathematical model suggests that following a live viral infection, virus-specific CD8 T cell population expands at a substantial rate, ρ_*L*_ = ρ + *m_LB_* = 1.4 day^−1^. Thus, our analysis provides minimal estimates of the expansion rates of virus-specific CD8 T cell populations in humans. Additional analysis also demonstrates that the estimated precursor frequency *E*_0_ is proportional to the frequency of naive T cells in the SLOs, with the proportionality coefficient being dependent on the rates of lymphocytes migration in and out of circulation (results not shown).

If expansion kinetics of CD8 T cell responses in humans is indeed much slower than that in mice, it remains unclear why this is the case. Many processes occur more rapidly in mice than in humans (e.g., heartbeat), so differences could arise due to differences in the metabolic rates (Demetrius, 2005). Precise reasons for differences in immunological response kinetics, however, remain to be investigated. Analysis of the kinetics of CD8 T cell response in major SLOs and the blood to a local infection of mice could provide valuable information on the processes impacting accumulation and loss of antigen-specific CD8 T cells in the circulation.

Despite slow expansion of the YFV-specific CD8 T cell response, the rise in the frequency of antigen-specific T cells was predictive of the clearance of YFV in circulation. However, since we analyzed average viral load data from multiple individuals it remains to be determined if expansion of virus-specific T cell population in individual subjects is predictive of viral clearance in the same subjects. It is important to note that in our analysis of the viral load data we ignored a decline in the amount of the virus found at day 2 post infection. The initial rise and decline of the virus could be a classical example of primary viremia when the virus spreads from the initial site of infection systemically. Primary and secondary viremia are well documented features of cytomegalovirus spread (Gerna, 2012). Because we did not have viral load data for individual subjects and because our model was already sufficiently complex, we did not include additional details for the viral spread within the host generating primary and secondary viremia if such phenomenon truly exists for YFV.

The correlation between expansion of YFV-specific CD8 T cell response and viral clearance in vaccinated individuals does not imply that T cells necessarily contribute to viral control. In fact, recent work suggests that T cell responses are not responsible for clearance of the YFV-based vaccine against dengue fever (Slifka, 2014). ChimeriVax is a vaccine in which YF-17D is used as a vector and in which YFV envelope gene has been replaced with dengue virus envelope (Slifka, 2014). The dynamics of this live dengue vaccine virus was similar in naive individuals or in hosts with pre-existent immunity to YFV (Guirakhoo et al., 2006) suggesting limited role of T cell responses to internal YFV proteins in control of virus replication (Slifka, 2014). Yet, viremia of the vaccine strain in immunized individuals was low and of a very short duration (1–2 days) which is in contrast to viremia observed in individuals exposed to YF-17D vaccine (Figure 4). In one well documented example vaccine-induced T cells are able to control and in many cases clear SIV infection but clearance often takes several weeks (Hansen et al., 2013). This result suggests that impact of virus-specific T cell responses on viral replication could be more pronounced for infections of longer duration which is consistent with the idea that vaccine-induced CD8 T cells may not be able to prevent infections (Slifka and Amanna, 2014).

It is interesting to note that in LCMV-infected mice, the timing of viral clearance is well correlated with the time when virus-specific CD8 T cell responses reach their peak suggesting that constant exposure to the antigen may be needed to sustain expansion of the T cell response (De Boer et al., 2001; Shaulov and Murali-Krishna, 2008). However, in YFV-infected individuals the virus is cleared from circulation several days before T cells are predicted to stop dividing. This may either suggest that viral antigens are still persistent in tissues to allow expansion of the T cell populations or that CD8 T cell responses are “programmed” in humans by the early exposure to the infection (Kaech and Ahmed, 2001). Further studies will be needed to discriminate between these alternative hypotheses.

Our analysis provides basic estimates for the rate of expansion of antibody-producing cell responses following exposure to VV and the rate of antibody production by these cells. The basic model in which virus-specific antibodies found in circulation are due to production by ASCs in circulation can readily explain the available experimental data. More realistic mathematical models that include change in the rate of antibody production by ASCs with time or migration of ASCs to the bone marrow, did not improve the quality of the model fit to data. We hypothesize that this occurred because only few measurements over the course of 3 months were available for the analysis. Also, it remains to be determined if our estimated rate of antibody production by ASCs are biologically realistic and consistent with what has been measured *in vivo* or *vitro* in several previous studies (Nossal and Makela, 1962; Salmon and Smith, 1970; Hiramoto et al., 1972b,a; Conrad and Ingraham, 1974; Hibi and Dosch, 1986). It should be noted, however, that previous studies vary dramatically on the estimates of the rate of production of immunoglobulins by antibody-secreting cells ranging from 300-500 molecules per second (Hiramoto et al., 1972a) to 12,000 and 30,000 molecules per second (Conrad and Ingraham, 1974; Hibi and Dosch, 1986). Our re-analysis of data from the most recent study suggests that plasma cells from trouts make 37 − 150 × 10^3^ IgG molecules per second (Bromage et al., 2009). The reasons for such large discrepancy in estimates are not known and could arise from different methods employed to estimate the rate of antibody production. To provide accurate estimates of the rate of antibody production by antibody-secreting cells future experimental studies should attempt to measure the total number of antibody-producing cells in circulations and in tissues (e.g., bone marrow) and to convert the titers of virus-specific antibodies to the actual concentration of the antibody molecules in circulation.

Our prediction on the kinetics of antibody production by ASCs is based on the assumption that VV-specific antibodies have a half-life of 2 weeks. We could not find a study that directly measured the half-life of VV-specific antibodies, and studies in mice, monkeys, and humans suggested a wide ranges of half-lives of IgG molecules ranging from 1 to 5 weeks (Vieira and Rajewsky, 1986, 1988; Slifka et al., 1998; Alyanakian et al., 2003; Salfeld, 2007; Pegu et al., 2014).

Allowing for a longer half-life of VV-specific antibodies (smaller value for δ_*a*_) allowed for a better description of the long-term dynamics of antibodies in the basic model (results not shown). This suggests that if VV-specific antibodies have a relatively high half-life, all antibodies found in circulation within 90 days post vaccination are likely to come from ASCs in circulation making antibodies at a constant rate, and evidence for alternative models is very weak. The antibody half-life also influenced the estimate of the antibody production rate by ASCs suggesting that the rate of loss of virus-specific antibodies is important in calculating how many antibody molecules one ASC makes per unit of time *in vivo*.

Our mathematical modeling-assisted analysis illustrates how simple kinetic data may provide important insights into dynamics of T cell responses to viruses, the mechanisms of viral control by T cells, and into how virus-specific antibodies are produced following vaccination. Our work also highlights problems with existing data which do not allow to discriminate between alternative models. Future experimental studies involving vaccination of human volunteers may benefit from more frequent measurements of the immune responses and from detailed mathematical analyses; such joint ventures between models and data are likely to generate novel insights into mechanisms leading to more efficient vaccines.

### Conflict of interest statement

The authors declare that the research was conducted in the absence of any commercial or financial relationships that could be construed as a potential conflict of interest.

## Abbreviations

YFV: yellow fever virus
VV: vaccinia virus
LCMV: lymphocytic choriomeningitis virus
SIV: simian immunodeficiency virus
NLME: non-linear mixed effects
NLS: non-linear least squares
RU: relative units
ASCs: antibody-secreting cells.
