# Supplementary material for: Mathematical modeling provides kinetic details of the human immune response to vaccination
Source: Front Cell Infect Microbiol. 2015 Jan 9;4:177. doi: 10.3389/fcimb.2014.00177 (PMC4288384; doi:10.3389/fcimb.2014.00177)
Supplement: Supplementary file 1 [file DataSheet1.PDF]

Supplement

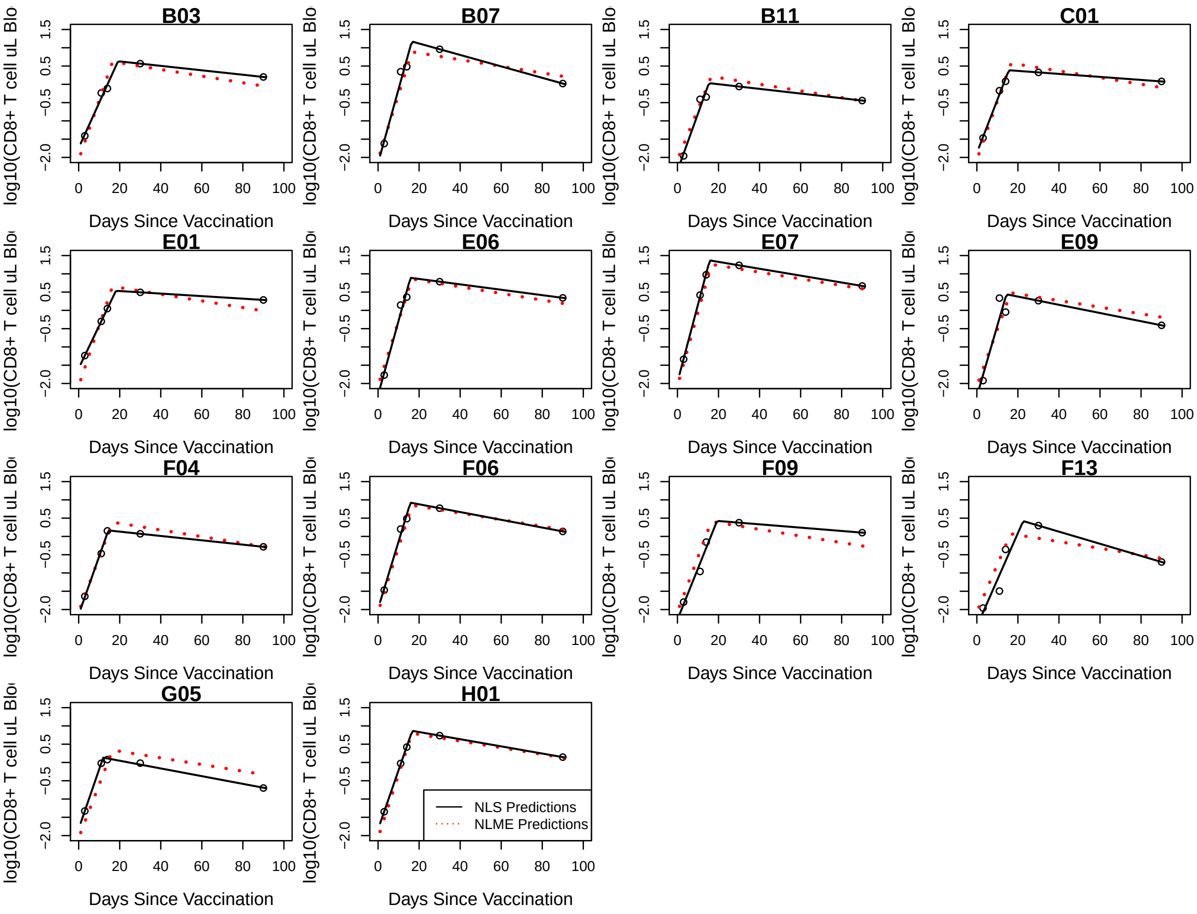

Figure S1: Predictions of the basic mathematical model on the CD8 T cell dynamics found using NLS and NLME methods. The model (eqn. (1) in the Main text) was fitted to experimental data using nonlinear least squares (NLS) or nonlinear mixed effects (NLME). Parameters of the model are given in Table 1 in the Main text (NLS) or in Table S1 in Supplement (NLME).

Patient	E_0, %	ρ, day^{-1}	T_{off}, day	δ_E, day^{-1}	$E_{\max}$, %
A02	0.0086	0.31	16.5	0.021	1.35
A03	0.0086	0.35	16.5	0.021	2.89
B03	0.0086	0.37	16.5	0.021	4.17
B04	0.0086	0.33	16.5	0.021	1.94
B07	0.0086	0.41	16.5	0.021	7.72
B10	0.0086	0.37	16.5	0.021	3.54
B11	0.0086	0.32	16.5	0.021	1.64
B15	0.0086	0.37	16.5	0.021	3.8
B17	0.0086	0.34	16.5	0.021	2.4
C01	0.0086	0.37	16.5	0.021	3.8
E01	0.0086	0.38	16.5	0.021	4.51
E05	0.0086	0.35	16.5	0.021	2.99
E06	0.0086	0.41	16.5	0.021	7.23
E07	0.0086	0.46	16.5	0.021	18.13
E09	0.0086	0.36	16.5	0.021	3.04
F04	0.0086	0.34	16.5	0.021	2.45
F06	0.0086	0.41	16.5	0.021	7.13
F09	0.0086	0.35	16.5	0.021	2.56
F10	0.0086	0.37	16.5	0.021	3.95
F13	0.0086	0.3	16.5	0.021	1.18
G05	0.0086	0.34	16.5	0.021	2.18
H01	0.0086	0.4	16.5	0.021	6.3
Average	0.0086	0.36	16.5	0.021	4.19
Stdev	0	0.038	0	0	3.59

Table S1: Estimates of the parameters of the mathematical model (eqn. (1) in the Main text) fitted to the data on the kinetics of YFV-specific CD8 T cell response using NLME package in R.
